# Dermatofibrosarcoma Protuberans of the Breast: A Clinicopathological Study of a Rare Cutaneous Low-Grade Sarcoma

**DOI:** 10.7759/cureus.76786

**Published:** 2025-01-02

**Authors:** Fatima Safdar, Nasir Ud Din, Abida K Sattar, Syeda Samia Fatima, Romana Idress

**Affiliations:** 1 Department of Pathology and Laboratory Medicine, Aga Khan University Hospital, Karachi, PAK; 2 Department of Pathology and Laboratory Medicine, Aga Khan University, Karachi, PAK; 3 Department of Surgery, Aga Khan University Hospital, Karachi, PAK

**Keywords:** breast, dermal lesion, dermatofibrosarcoma protuberans, low grade, sarcoma

## Abstract

Background: Dermatofibrosarcoma protuberans (DFSP) of the breast is a rare entity. It is a slow-growing soft tissue tumor of low to intermediate grade. The risk of metastasis is low, but its likelihood of local recurrence is significant.

Objective: Our study aims to present the clinical and histological features of DFSP of breast and follow-up.

Material and methods: Patients with histologically proven DFSP between 01 January 2010 and 31 October 2023 were identified from a prospectively maintained pathology database. Two senior pathologists reviewed the clinical data and histological slides, and a follow-up was obtained.

Results: Twenty-six cases of DFSP breast were diagnosed between 01 January 2010 and 31 October 2023. Out of 26, 10 (38.5%) were male and 16 (61.5%) were female. The mean age of presentation was 37.2 years in females and 40.7 years in males. The mean tumor size in females was 4.7 cm and in males was 5.4 cm. Histologically, the 15 DFSP cases (58%) showed spindle cells arranged in a storiform pattern with honeycomb-type fat infiltration. Fibrosarcomatous transformation was noted in 11 (42%) cases comprising a fascicular pattern. The median follow-up period was 6.1 years. Three (12%) patients experienced recurrence. No recurrence was observed in 23 (88%) patients with complete surgical resection.

Conclusions: We present the largest series of breast DFSP. The recurrence rate of 12% aligns with the DFSP of other common sites. Fibrosarcomatous transformation in breast DFSP (42%) is higher as compared to DFSP in other common locations and its long-term clinical behavior cannot be reliably predicted due to lack of long-term follow-up.

## Introduction

Dermatofibrosarcoma protuberans (DFSP) is a rare soft-tissue sarcoma accounting for 1% to 6% of all soft-tissue sarcomas [1-3]. DFSP is a low-to-intermediate-grade soft tissue tumor that grows slowly and has a high rate of local recurrence but a low risk of metastasis [3,4]. It is characterized by translocation of the *COLlAl and PDGFRB *genes [2]. DFSP can involve any part of the body, with a predilection for the trunk and limbs followed by the genital tract, and rarely the breast. It occurs most frequently between the second and fifth decades of life [1,4,5]. Clinically, it presents as a slow-growing, painless cutaneous mass. It appears as a small, blue-reddish subcutaneous lump that invades the surrounding fascia and subcutaneous tissue, growing slowly [2].

Microscopically, DFSP is usually characterized by spindle cells arranged predominantly in a storiform pattern with little nuclear pleomorphism and mitosis. Approximately 85-90% of DFSPs are low-grade, while the remaining cases have a high-grade sarcomatous component, which is typically identified as fibrosarcoma and considered an intermediate-grade sarcoma [6,7]. Fibrosarcomatous DFSP is a rare pathologic variant with more aggressive infiltrative growth and a higher rate of metastasis. Microscopically, fibrosarcomatous DFSP shows fascicular to herringbone growth with a higher degree of pleomorphism and mitotic activity [8]. Immunohistochemically, CD34 is positive in 90% of the cases and S100, actin, and desmin are negative [2].

Due to the rarity of breast DFSP cases, the data is still under study [4,9,10]. We present the largest case series of breast DFSP, including clinicopathological features, and follow-up of patients. The objective of our study is to present the clinical and histological features of DFSP of the breast. Follow-up of patients including history of any post-operative therapy and recurrence was taken.

This article was previously presented at the 111th United States and Canadian Academy of Pathologists (USCAP) Annual Meeting, held on March 19-24, 2022, and its abstract was published [11].

## Materials and methods

Study design, inclusion, and exclusion criteria

This is a descriptive observational study. All patients diagnosed with DFSP of the breast at the Aga Khan University Hospital, Pathology and Laboratory Department, Karachi, Pakistan, between 01 January 2010 and 31 October 2023 were searched electronically in the Institutional Integrated Laboratory Management System (ILMS). Inclusion criteria were patients (irrespective of age and gender) diagnosed with DFSP breast between 01 January 2010 and 31 October 2023. Exclusion criteria were poor quality or unavailable slides and blocks for histopathological review.

Data collection

The study was approved by the Aga Khan University Ethics Review Committee (ERC number: 2022-6912-21068, Approval date: 22-Mar-2022). Patient information such as demographics including age, gender, tumor size, and surgical procedure was extracted from the histopathology reports. Hematoxylin and eosin stained (H&E) and immunohistochemistry (IHC) stained slides of these cases were retrieved from the archives and were reviewed by two pathologists, one with experience in breast pathology and one with soft tissue pathology experience. A record in the form of proforma was made, which included mitotic count, fibrosarcomatous transformation, and skin ulceration. Ancillary studies, including immunohistochemical stains, were also reviewed. Additionally, relevant clinical history including presenting complaints, subsequent treatment, and follow-up was obtained after telephonic consent and communication from patients/attendants.

Statistical analysis

Descriptive statistical analysis was performed using Stata version 17 (StataCorp LLC, College Station, USA). Frequencies and percentages were obtained for all the categorical variables including gender. Measures of central tendency and variability were obtained for continuous data such as age and tumor size.

## Results

**Figure 1:**
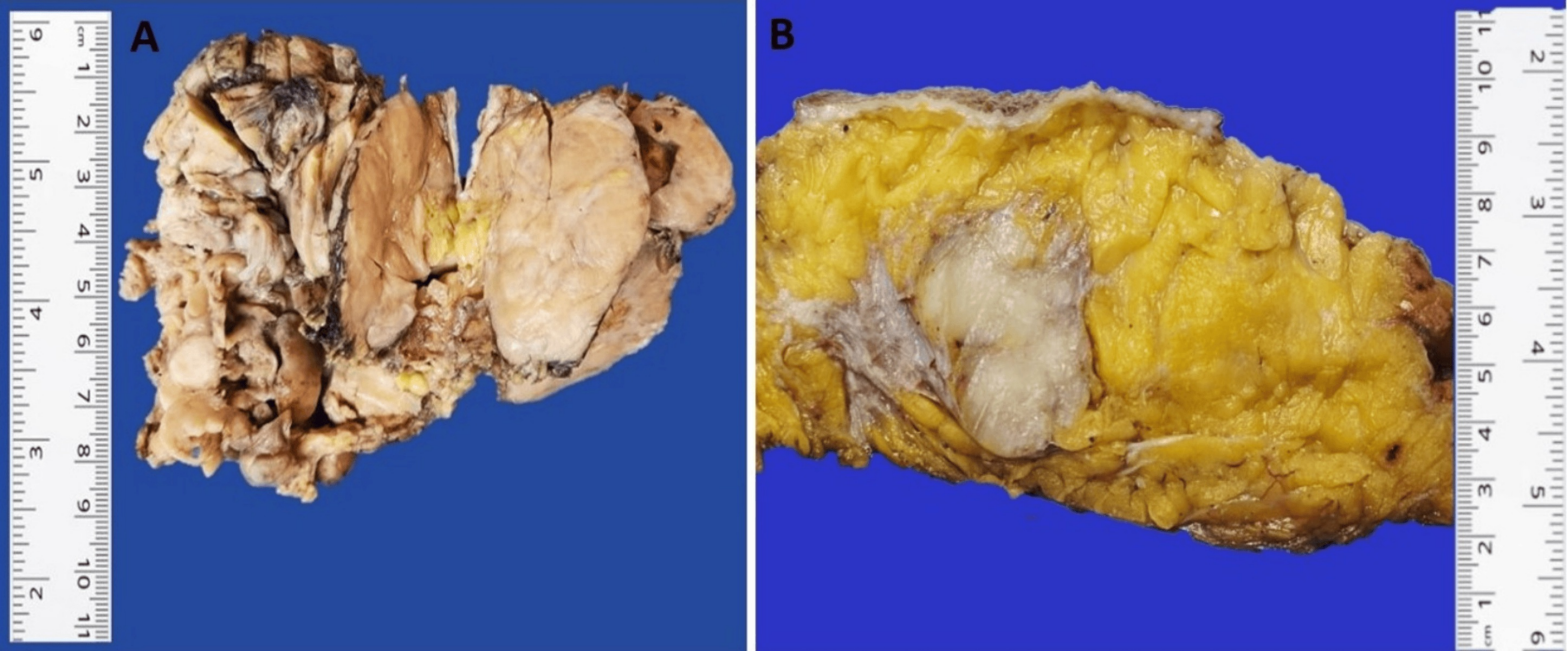
(A) The image shows a multilobulated lesion ulcerating the overlying skin. (B) The image shows a circumscribed, multinodular, tan-white lesion.

A total of 26 cases of DFSP of the breast were diagnosed between 01 January 2010 and 31 October 2023. Out of 26, 10 (38.5%) were male and 16 (61.5%) were female. The mean age of presentation was 37.2 years (range 12 to 54 years) in females and 40.7 years (range 25 to 70) in males. The mean tumor size in females was 4.7 cm (range 1.0 to 18 cm) and in males was 5.4 cm (range 1.0 to 20 cm) (Table 1). In 19 out of 26 excisional specimens, the nipple and areola were present in six (32%) specimens, and skin ulceration was seen in six (23%) cases (Figure 1A). The cut surface was firm, white, and multilobulated (Figures 1A-1B). Most cases (92%) were operated and managed at other institutions.

**Table 1:** Descriptive statistics The data provides a detailed summary of the demographic characteristics and key parameters of the patients. Out of a total of 26 patients, 10 patients (38.5%) were male, and 16 patients (61.5%) were female. The skin ulceration was identified in six patients (23.1%), absent in 16 patients (61.5%), and not applicable (as skin was not present in the specimen) in four patients (15.4%) and fibrosarcomatous transformation was identified in 15 patients (58%) and absent in 11 patients (42%). The mean age is 38.518 years with a standard deviation (SD) of 11.659, while the mean tumor size is 4.95 cm with an SD of 5.572.

		N	Percentage (%)
Sex	Male	10	38.5
	Female	16	61.5
Skin ulceration	Present	06	23.1
	Absent	16	61.5
	Not applicable	04	15.4
Fibrosarcomatous transformation	Present	15	58
	Absent	11	42
		Mean	SD
Age	-	38.518	11.659
Tumor size	-	4.95	5.572

**Figure 2:**
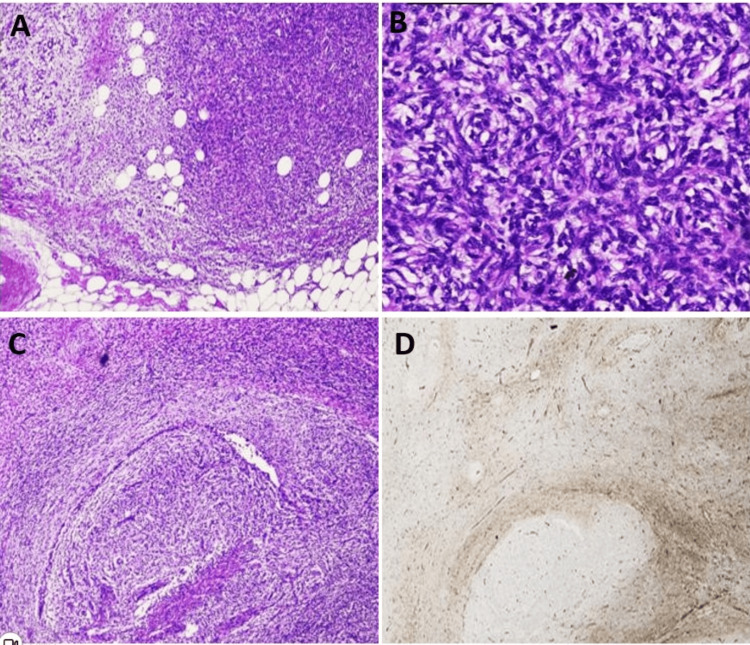
(A) Proliferation of spindle cells with characteristic honeycomb appearance and breast parenchyma at the periphery (H&E, X10). (B): Classic storiform pattern of dermatofibrosarcoma protuberans (DFSP) (H&E, X20). (C): DFSP transition with fibrosarcomatous areas (H&E, X20). (D): Loss of CD34 in fibrosarcomatous areas (H&E, X20). H&E: hematoxylin and eosin; DFSP: dermatofibrosarcoma protuberans

Histologically, 15 DFSP cases (58%) showed spindle cells arranged in a storiform pattern with honeycomb-type fat infiltration (Figures 2A-2B). Fibrosarcomatous transformation was noted in 11 (42%) cases comprising a fascicular pattern along with residual foci of the storiform pattern (Figure 2C). The mean mitotic count in 15 DFSP cases was 4.3 per high-power field (HPF), with a range of 2 to 8/HPF. In contrast, the mean mitotic count in 11 fibrosarcomatous DFSP cases was 20.6 per HPF, with a range of 7 to 72/HPF. The breast ducts and acini were noted in all six excisional specimens exhibiting nipple and areola. On IHC, 15 out of 26 DFSP cases (58%) showed diffuse strong CD34 positivity. In contrast, 9 out of 11 fibrosarcomatous DFSP cases exhibited weak staining in the fascicular areas, while two cases were completely negative for CD34 (Figure 2D).

The median follow-up period was 6.1 years (range 4 months to 14 years). All patients were alive and healthy at the last follow-up. Three (12%) patients experienced recurrence after a mean follow-up of 6.7 years (Table 2). Tumors were incompletely excised in these three patients and resection margin was involved in these three cases. In 23 (88%) patients with complete surgical resection, no recurrence was observed.

**Table 2:** Details of cases with recurrence The data provides a detailed summary of the demographic characteristics and key parameters of the patients with recurrence. Out of three patients, one patient (33.3%) was male, and two patients (66.7%) were female. The skin ulceration and fibrosarcomatous transformation was absent in all three patients (100%). The mean age is 29 years with a standard deviation (SD) of 5.291, while the mean tumor size is 1.5 cm with an SD of +0.

		N	Percentage (%)
Sex	Male	01	33.3
	Female	02	66.7
Skin ulceration	Present	00	00
	Absent	03	100
Fibrosarcomatous transformation	Present	00	00
	Absent	03	100
		Mean	SD
Age (years)	-	29	5.291
Tumor size (cm)	-	1.5	+0

## Discussion

DFSP is a low-to-intermediate-grade soft tissue tumor that typically develops in the dermis and extends to subcutaneous tissues and muscles [7,10]. It accounts for <0.1% of all malignancies and about 1% to 6% of all soft-tissue sarcomas [2,6,11]. The trunk is the most common site involved (50-60%), followed by limbs (25%), head and neck (10-15%), and rarely breast [7,12,13].

The molecular studies showed translocation involving chromosomes 17q22 and 22q13 leading to a fusion protein that promotes the continuous activation of PDGF receptor beta (PDGFR-beta) protein-tyrosine kinase, found in more than 90% of DFSP. It provides the rationale for the use of imatinib mesylate (IM) and other tyrosine kinase inhibitors (TKIs) currently used in the treatment of DFSP [14,15]. Though Wang et al. published a single review in 2020, there are very few cases in the literature, most of them as case reports [2].

Breast DFSP presents a well-circumscribed, oval-shaped lesion that is skin-based or intra-mammary without speculation or microcalcification on mammography. Due to their lack of specificity, these mammography results can be mistaken for benign breast tumors including fibroadenoma, sebaceous cysts, or abscesses. Owing to its infiltrative nature, DFSP frequently spreads beyond the clinical margin [7-9].

Histologically, DFSP exhibits monomorphous spindle-shaped cells on a background of fibrous stroma arranged in a storiform pattern, with few mitotic figures and mild nuclear pleomorphism. It infiltrates subcutaneous adipose tissue, exhibiting a distinctive "honeycomb" pattern. Immunohistochemically, it is diffusely and strongly CD34 positive as seen in our cases (58%), and negative for keratin, smooth muscle actin (SMA), desmin, and S100 [7,8,12,13]. DFSP has several subtypes including pigmented (Bednar), atrophic, sclerosing, fibrosarcomatous, giant cell fibroblast-like, granular cell variant, and myxoid DFSP. Fibrosarcomatous dermatofibrosarcoma protuberans (DFSP-FS) is an aggressive subtype of DFSP, characterized by intermediate-to-high-grade sarcoma, with cytological atypia and increased mitotic rates as compared to DFSP. IHC also revealed a decreased level of CD34 expression in DFSP-FS [1,8]. In our study of 9 out of 11 fibrosarcomatous DFSP cases, CD34 staining was weak in the fascicular areas and completely negative in two cases.

Phyllodes tumor of the breast, nodular fasciitis, classic-type myofibroblastoma, solitary fibrous tumor, and desmoid-type fibromatosis are among the differential diagnoses for DFSP in the breast [3,13]. Nodular fasciitis presents clinically as a rapidly growing mass and is composed of fibroblasts and/or myofibroblasts with no significant cytological atypia or pleomorphism. They are positive for SMA; can have focal desmin positivity; and are negative for keratins, CD34, S100, and beta-catenin. Myofibroblastomas are typically slow-growing and exhibit short intersecting fascicles with interspersed variably hyalinized collagen bundles. In contrast to DFSP, they are typically positive for desmin and CD34, along with the estrogen receptor, progesterone receptor, and androgen receptor. Breast fibromatosis exhibits a more infiltrative pattern, entraps lobules and mammary ducts, and is nuclear positive for beta-catenin. Phyllodes tumors have a biphasic growth pattern with both stromal and epithelial components. It is possible to confuse fibrosarcomatous transformation of DFSP with significant stromal overgrowth with malignant phyllodes tumors. In these cases, immunohistochemical stains are not helpful; instead, the identification of remaining epithelial structures through thorough sampling is necessary for the diagnosis of phyllode tumors [15].

Wide local surgical excision (WLE) is the standard treatment for localized DFSP tumors. The safe resection margin is still uncertain, although an excision of at least 2 to 3 cm, including skin, subcutaneous tissue, and fascia, is widely recognized because histologically tumor-free margins differ greatly from clinically free ones. Mohs Micrographic Surgery (MMS) produces improved tridimensional margin control by allowing the extent of excision to be tailored to the microscopic extent of the tumor [1]. When compared to WLE, MMS provides comparable but reduced local recurrence rates. The prognosis of the disease is not improved by elective lymph node excision [1-4,16-21]. Three patients who had recurrence had margins involved at the time of initial biopsy, in our study. The remaining patients had a mean distance of 1.5 cm from the resection margin.

The response of DFSP to adjuvant treatment including chemotherapy and radiotherapy is studied by various studies. For recurrent tumors or unresectable residual lesions, adjuvant radiotherapy is recommended to control the progression of the disease. Furthermore, Chen et al. [22] showed that adjuvant radiation therapy might be taken into consideration for all patients undergoing surgical excision, regardless of the surgical margin, in a meta-analysis involving 167 DFSP patients treated with adjuvant radiation [3,4,12]. Furthermore, studies showing a decrease in DFSP tumor size and the response rates of neoadjuvant imatinib are reported. In a phase II trial with two-month neoadjuvant imatinib therapy, 36% of patients showed a clinical response [15]. In a separate trial, Han et al. [20] noted clinical response rates with an average preoperative tumor size decrease of 36.9% [9,20,21]. No patient received any adjuvant therapy in our case series.

DFSP has a low propensity for metastasis and a significant probability of local recurrence. Although late recurrences have also been seen, most local recurrences occur within three years after surgery. Therefore, it is advised that long-term follow-up should be done for breast DFSP cases. In our series, three individuals had recurrences, with a mean interval of about 10 years. The poor prognostic characteristics of DFSP include advanced age, a large tumor size, the DFSP-FS subtype, and inadequate resection [4]. Rarely, 1-4% of systemic metastasis is seen after several local recurrences. Hematogenous metastases most frequently occur in the lungs [2,7,8,16]. In our study, metastasis to systemic sites was not reported in any patient. Breast DFSP is difficult to diagnose because of its rarity, capacity to resemble a range of benign and malignant breast diseases, and increased readiness for precise identification and treatment [2].

The main limitations of this study include the single-center design and small sample size. A multicenter study would increase the sample size and add the experience of more histopathologists.

## Conclusions

We present the clinicopathological features of a large cohort of breast DFSP. The recurrence rate was 12%, which is similar to the recurrence rate seen in DFSP occurring at more common sites. Inadequate surgery with margin involvement was the main cause of recurrence. The fibrosarcomatous transformation in breast DFSP (42%) was higher as compared to DFSP in other common locations including the trunk and proximal extremity. The behavior of fibrosarcomatous DFSP in the breast cannot be reliably assessed as most of these cases have short follow-ups. Long-term follow-up and other clinical studies including molecular studies in the future are recommended.
